# Sexual Dysfunction and Associated Psychiatric Comorbidities Impacting Quality of Life in Epilepsy: A Review of Literature

**DOI:** 10.7759/cureus.51266

**Published:** 2023-12-29

**Authors:** Usha Kasar, Amitabh K Dwivedi

**Affiliations:** 1 Department of Occupational Therapy, Maharaj Vinayak Global University, Khorameena, IND; 2 Department of Occupational Therapy, Jagadguru Sri Shivarathreeshwara (JSS) Medical College, JSS Academy of Higher Education and Research, Mysuru, IND

**Keywords:** anxiety and depression, antiepileptic drugs, quality of life, epilepsy, sexual dysfunction

## Abstract

Introduction: Epilepsy is caused by dysfunction in the brain, which is characterized by an enduring disposition for unprovoked seizures that reoccur often, along with the neurological, cognitive, psychological, and social effects of the condition, which are often managed with prolonged and sometimes lifelong medications that involve antiepileptic drugs (AEDs). To confirm the diagnosis of epilepsy, at least two unprovoked seizures occurring greater than 24 hours apart should be reported. This illness affects both children and adults. Patients with epilepsy are more likely to experience sexual dysfunction compared to the general population, which often leads to a poor quality of life. The pathophysiology involved consists of the impact of epilepsy and AEDs on the control of the hypothalamic-pituitary-gonadal axis, consequently resulting in a high risk of developing testicular or ovarian dysfunction, leading to sexual dysfunction in epilepsy patients.

Aim: This review aims to highlight various studies focusing on the association of developing the risk of sexual dysfunction with psychiatric comorbidities, mainly depression and anxiety, that impact the quality of life in epilepsy patients.

Methodology: The search methodology involved articles from the Google Scholar and PubMed databases published between 2018 and 2023. "Sexual dysfunction", "epilepsy", "depression", "anxiety", and "quality of life" were the keywords used to search the articles. The Boolean operator "AND" and "OR" were used in between the keywords used. Following this, a total of 15 articles were included in the review based on the inclusion and exclusion criteria.

Conclusion: The review concluded that epilepsy patients are often affected by sexual dysfunction along with associated psychiatric comorbidities that mainly involve depression and anxiety, consequently impacting their quality of life, as demonstrated by various studies. Sexual dysfunction is a common yet underdiagnosed condition in epilepsy patients due to the stigma attached to it. Hence, neurologists must keep a high index of suspicion for this problem. Furthermore, screening and monitoring for sexual dysfunction should be added to the usual epilepsy work-up.

## Introduction and background

Epilepsy is a brain disorder marked by an enduring disposition for unprovoked seizures that reoccur often, as well as the neurological, cognitive, psychological, and social effects of the condition. The diagnosis of epilepsy requires at least two unprovoked seizures occurring greater than 24 hours apart. A syndrome is defined as a seizure associated with abnormal investigations that occur together in a recognizable pattern [1]. It usually includes more than one type of epilepsy that is often managed by medications, which are generally required throughout the lifetime. It generally affects both adults and children and affects one in every 26 people at some point in their lives [2]. This condition often necessitates prolonged, sometimes lifelong, treatment with antiepileptic drugs (AEDs). Additionally, despite numerous new AEDs continuously being developed, nearly 40% of all epilepsy patients still experience uncontrollable seizures [3-6]. In India, there are 5.5 million people with epilepsy, which is one-eighth of all epilepsy patients in the world [2].

The occurrence of sexual hormone abnormalities is often associated with AED use, which mainly involves carbamazepine, phenytoin, and phenobarbitol, which induce liver enzymes that lead to suppression of gonadal testosterone synthesis and disturbance of other peripheral sex steroid hormones [7-10]. The control of the hypothalamic-pituitary-gonadal axis is directly impacted by epilepsy and AEDs, placing epilepsy patients at a high risk of developing testicular or ovarian dysfunction. Due to interruptions in the medial temporal lobe and sex steroid hormone metabolism, both female and male epilepsy patients may have dysfunction in central control, leading to a decrease in androgen, consequently leading to sexual dysfunction. According to research findings, the percentage of sexual dysfunction in epilepsy patients varied between 23% and 60% in female patients and between 38% and 71% in male patients. Among male epilepsy patients, the most common complaints were hyposexuality, erectile dysfunction (ED), and orgasmic sexual issues. Whereas in female epilepsy patients, orgasmic dysfunction, lack of sexual desire, less sexual arousal, and dyspareunia were the prominent sexual problems [7-10]. In males, sexual dysfunction was mainly caused by a hormonal imbalance that involved an increase in follicle-stimulating hormone, estrogen, and sex hormone-binding globulin and a decrease in dehydroepiandrosterone and the free androgen index. Additionally, concomitant neuropsychiatric illnesses, including anxiety and depression, that are known to exacerbate sexual dysfunction can be brought on by epilepsy [6-10].

In comparison to the general population, sexual dysfunction is more prominent in epilepsy patients [11-13]. It has significant psychological, biological, and social ramifications and often leads to a poor quality of life. Sexual dysfunction is an important yet underdiagnosed comorbidity among epilepsy patients. It is difficult to diagnose because of the societal taboo attached to it. The most common problems encountered by these patients involve orgasmic disorders, dyspareunia, erectile dysfunction, decreased sexual desire, early climax or ejaculation, and vaginal dryness. The epilepsy patients are often not forthcoming with these complaints and, at times, think sexual dysfunction to be unrelated to epilepsy [10-13]. Therefore, this review highlights various studies focusing on the association of developing the risk of sexual dysfunction with psychiatric comorbidities, mainly depression and anxiety, that impact the quality of life in epilepsy patients.

## Review

Search methodology

The search methodology involved articles from the Google Scholar and PubMed databases published between 2018 and 2023. "Sexual dysfunction", "epilepsy", "depression", "anxiety", and "quality of life" were the keywords used to search the articles. The Boolean operators "AND" and "OR" were used in between the keywords used. The inclusion criteria consisted of articles giving information regarding sexual dysfunction and associated psychiatric comorbidities involving anxiety and depression and providing information on the quality of life in epilepsy patients with full-text availability in the English language. The articles do not providing brief descriptions regarding the sexual dysfunction in epilepsy patients, associated psychiatric comorbidities, and information related to the quality of life in epilepsy patients in the English language, with the non-availability of full-text protocols, and editorials, and studies performed on animals were excluded from the study. 

**Figure 1:**
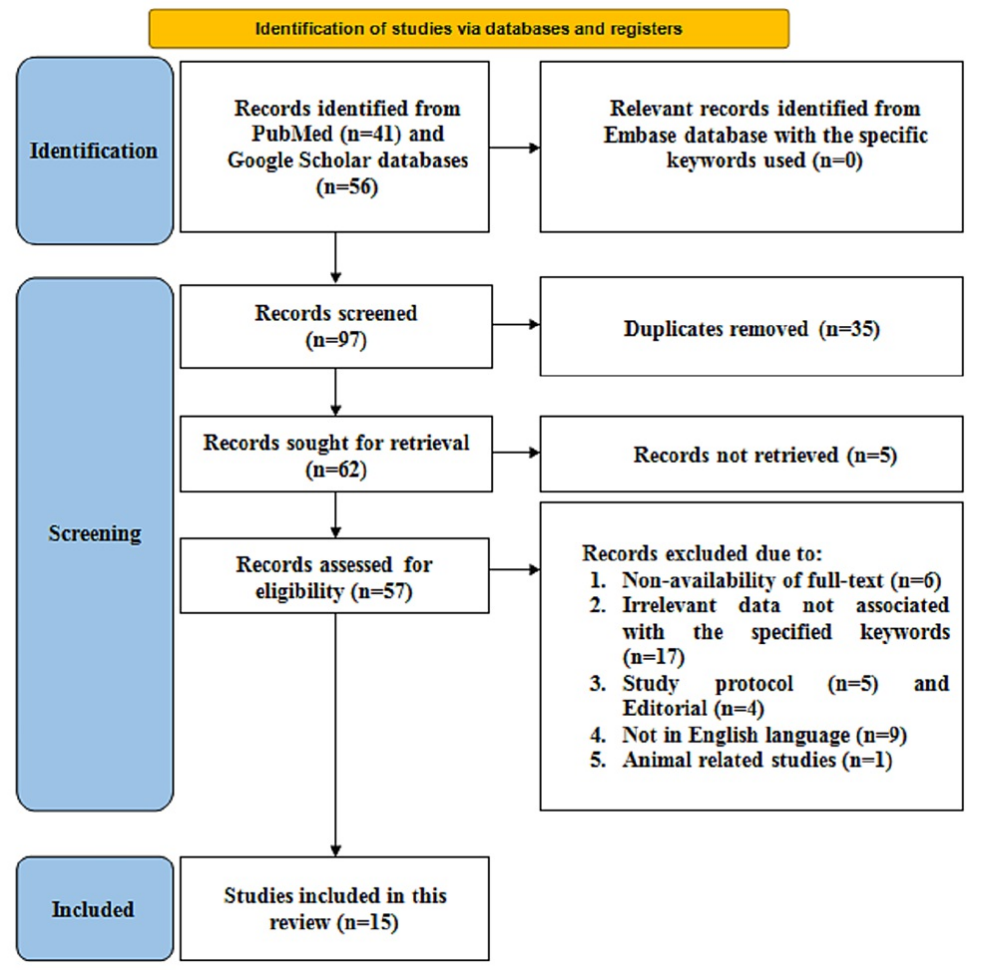
Search strategy in accordance with PRISMA guidelines PRISMA: Preferred Reporting Items for Systematic Review and Meta-Analysis

For the initial selection of studies, on the PubMed database, 41 articles were found, and 56 articles in the Google Scholar database, summarizing 97 articles. The relevant records that were identified from the Embase database with the specific keywords "sexual dysfunction," "epilepsy," "depression," "anxiety," and "quality of life," along with the Boolean operators "AND" and "OR," were found to be zero (n=0) leading to the screening of an overall 97 articles. From the 97 articles screened, 35 duplicates were removed, after which 62 were sought for retrieval, which means the articles were again screened based on the keywords mentioned and by assessing the title and abstracts of the articles, of which five were not retrieved. Overall, 57 records were then assessed for eligibility, from which 42 articles were excluded, as six articles did not provide full-text availability, 17 articles demonstrated irrelevant data that was not associated with the specified keywords, five articles represented study protocols, four articles represented editorials, nine articles were not present in the English language, and one article was related to an animal study. After the exclusion of 42 articles, which was based on the inclusion and exclusion criteria, a total of 15 articles were included in this review (Figure 1). This review followed Preferred Reporting Items for Systematic Review and Meta-Analysis (PRISMA) guidelines for search strategy, as illustrated in Figure 1.

Data extraction

Table 1 provides a brief description of the data, which includes the author and year, the purpose of the study, the methodology, and the summary of the findings used in this evaluation.

**Table 1:** Summary of the studies included in this review MS: Multiple Sclerosis, CSFQ: Changes in Sexual Functioning Questionnaire, SF-36: Short Form 36 Health Survey, PCS: Physical Component Summary, MCS: Mental Component Summary, LiSat-11: Life Satisfaction-11, AEDs: Antiepileptic Drugs, SD: Sexual Dysfunction, ASEX: Arizonal Sexual Experience Scale, HAM-D17: Hamilton Rating Scale for Depression, QOLIE-31: Quality of Life Inventory Epilepsy-31, FSFI: Female Sexual Function Index, SAS: Self-rating Anxiety Scale, SDS: Self-rating Depression Index, MMAS: Morisky Medication Adherence Scale, NHS3: National Hospital Seizure Severity Scale, VPA: Valproate, OXC: Oxcarbazepine, IIEF-5: International Index of Erectile Function-5, DSMV: Diagnostic and Statistical Manual of Mental Disorder Fifth Edition, QOL: Quality of Life, ESI: Epilepsy Surgery Inventory, BDI: Beck Depression Scale, STAI-X: State-Trait Anxiety Inventory, PHQ-9: Physical Health Questionnaire-9, GAD-7: Generalized Anxiety Disorder-7, ODTA: Occupational Therapy Program with Drama Activities, GTCS: Generalized Tonic Clonic Seizures

Sr No.	Author and year	Aim of the study	Methodology and Results	Conclusion
1.	Casale M et al. (2021) [3]	The prevalence of psychiatric comorbidities and measures of sexual performance in males with and without epilepsy were analyzed.	To gather information about demographics, epilepsy history, the type of AEDs taken at the time of conception, comorbidities, and sexual health, 450 male patients with and without epilepsy completed questionnaires. It was observed that epilepsy patients (n=110) reported a higher rate of psychiatric comorbidities such as general anxiety disorder, bipolar disorder, depressive disorder, and suicidal ideation when compared to the control group (without epilepsy, n=110) with statistically significant results (p<0.001).	Men with epilepsy had a higher frequency of psychiatric comorbidities and decreased sexual performance, notably involving erectile dysfunction.
2.	Zhao S et al. (2019) [10]	This meta-analysis aimed to quantify the association between epilepsy and the risk of sexual dysfunction.	The PubMed, Embase, and Cochrane Library databases were systematically searched to identify the pertinent studies focusing on the association between epilepsy and sexual dysfunction. Nine studies were included, for a total of 1556 subjects and 599 cases of epilepsy. The results demonstrated that out of a total of nine studies, six reported female sexual dysfunction and three reported male sexual dysfunction, with statistically significant results (p<0.001).	Epilepsy was associated with an increased risk of sexual dysfunction in both genders. These findings imply that epilepsy has a potentially dangerous impact on sexual function, which physicians and patients should be aware of.
3.	Kumar DP et al. (2020) [13]	Estimated the proportion of sexual dysfunction among people with epilepsy and determined the associated demographic and clinical factors.	The outcome measures used were CSFQ, PHQ-9, and GAD-7. After screening 3225 epilepsy patients, 108 patients were recruited. Out of the total 108 patients, 65 had sexual dysfunction, 64 had depression, and 63 had anxiety. Sexual dysfunction demonstrated a significant association with depression (p=0.01) and anxiety (p=0.04).	The study concluded that 60% of people with epilepsy had SD, along with a proportion of depression and anxiety.
4.	Henning O et al. (2019) [14]	To study the occurrences of different sexual problems in epilepsy patients and compare them with the general population.	For the study, a total of 221 patients participated in a questionnaire survey on sexual function. The outcomes were compared with similar data on sexual function collected from 1671 adults in the general population. The maximum number of epilepsy patients reported orgasm, dyspareunia, erectile dysfunction, and feelings of sexual deviance.	Patients with epilepsy reported a significantly higher frequency of problems with orgasm, dyspareunia, erectile dysfunction, and feelings of sexual deviance. However, lower-sex desire, an early climax or ejaculation, and vaginal dryness were equally common in the general population. Feelings of sexual deviation were linked to lower quality of life in both sexes.
5.	Petersen M et al. (2020) [15]	The primary aim of this study was to describe the prevalence of sexual dysfunction in patients with epilepsy and MS and investigate whether there is an association between disease, sexual function, and physical and mental health.	A total of 414 patients were included in this descriptive cross-sectional study. The CSFQ cut-off score, SF-36, was divided into the PCS and MCS, and the LiSat-11 was used. The results reported that out of 414 patients, 258 (62%) had MS and 158 (38%) had epilepsy. It was found that 68% of women and 77% of men demonstrated sexual dysfunction.	The cohort of patients with MS and epilepsy had negatively affected sexual function. When sexual function was assessed by the CSFQ-14 in patients with MS and epilepsy, the only noticeable difference was in the frequency of desire, where a greater proportion of patients with epilepsy reported sexual dysfunction. Sexual function in men is linked to physical health, whereas in women, it is associated with physical as well as mental health.
6.	Tudor K et al. (2021) [16]	This study aimed to evaluate the relationship between epilepsy, AEDs, and SD and its association with quality of life and depressive symptoms.	This was a prospective study in which SD was evaluated using the internationally acclaimed questionnaire ASEX, which was successfully translated into Croatian and validated for this purpose. Depressive symptoms and quality of life were evaluated using the HAM-D17 and QOLIE-31. There were no significant correlations between the type of epilepsy and SD, nor between the AEDs (old generation vs. modern) and SD. Significant correlations were found between the SD and more pronounced depressive symptoms (p=0.003) and between the SD and a lower quality of life (p=0.001).	The study's findings indicate that around one-third of the patients in our group exhibit SD, which is comparable to the prior percentage of SD reported in the population sample. The symptoms of SD were observed to be more severe in women. While there was no significant link with the type of epilepsy or the use of AEDs, it was shown that SD symptoms were substantially correlated with older age, female gender, a lower quality of life, and depressive symptoms.
7.	Tao L et al. (2018) [17]	Aiming to assess the factors associated with sexual dysfunction in Chinese Han women with epilepsy	This cross-sectional study examined 112 married women with epilepsy and 120 healthy controls without epilepsy. The Chinese version of FSFI, the Chinese version of Zung SAS, SDS, the Chinese version of the revised MMAS-8, and the Chinese version of the NHS3. A high rate (70.5%) of sexual dysfunction was detected in women with epilepsy, compared to 24.2% in controls. Sexual dysfunction affected all dimensions: desire (85.7%), arousal (56.3%), lubrication (47.3%), orgasm (66.1%), satisfaction (58.9%), and pain (41.1%). Elevated rates of anxiety (40.2%), depression (33%), and poor medication adherence (31.3%) were also found in women with epilepsy.	The results demonstrated that a high rate of SD was detected in women with epilepsy. SD affected all dimensions involved, mainly desire, arousal, lubrication, orgasm, satisfaction, and pain. Elevated rates of anxiety and depression and poor medication adherence were also found in women with epilepsy.
8.	Guo Yi et al. (2021) [18]	This study is aimed at evaluating the effects of AEDs on sexual function, sperm quality, and sex hormones in young males with epilepsy.	Males with recently diagnosed epilepsy who were taking VPA and OXC were enrolled, and sex hormone levels, sperm quality, and the IIEF-5 were evaluated prior to and six months after treatment with VPA or OXC monotherapy. Forty-four young males with epilepsy (23 treated with VPA, 21 treated with OXC) and 30 age-matched healthy individuals were recruited for our study. The sexual function, sperm quality, marriage rate, and fertility rate of these young males with epilepsy were lower than those of healthy controls. Sperm quality was significantly reduced in young male patients after six months of VPA administration. The level of follicle-stimulating hormone (FSH) was increased in patients after OXC treatment. Meanwhile, sexual function and sperm quality were not affected.	In young males with epilepsy, sperm quality and sexual function were decreased. In young epileptic males, VPA may have a detrimental effect on sperm quality, while OXC has no significant impact on sexual function or sperm quality.
9.	Özek Ü et al. (2021) [19]	The study aimed to assess potential SD in patients with epilepsy who have no known psychiatric disorders or disabilities.	In this study, 48 patients with epilepsy and 69 healthy controls participated. The five components of sexual function were evaluated in both patients and controls using the ASEX, and probable SD was screened using the total ASEX score. The relationship between SD and demographic and clinical parameters was examined. In the patient group, 54% were female and 46% were male. The mean age was 39.3±10.4 years in female patients and 40.0±7.1 years in male patients. In female patients, seizures were classified as focal in 57.7% and generalized in 42.3%, while in male patients, seizures were classified as focal in 45.5% and generalized in 54.5%. The mean duration of the disease was 25.7 ± 11.7 years. Male patients with epilepsy had statistically lower ASEX ejaculation subscale scores compared to controls (p < 0.05). The ASEX score was not associated with the duration of the disease or the number of antiepileptic drugs used (p > 0.05).	Compared to controls, male patients with epilepsy demonstrated potential ejaculation dysfunction.
10.	Ogunjimi L et al. (2018) [20]	Studied SD among Nigerian women with epilepsy.	A descriptive study was carried out in which Zung SDS was used to assess mood, and SD was measured using the ASEX questionnaire. The frequency of clinically significant SD among FWE (35, 50.0%) was similar to that of controls with statistically insignificant results (p=0.173). The mean ASEX score was higher in FWE than in controls (p = 0.009).	The frequency of clinically significant SD among females with epilepsy was similar to that of controls. However, compared to controls, the mean ASEX score was greater in female epilepsy patients, as higher scores were demonstrated in all domains of the DSM-V. When compared to women with non-lesional epilepsy, females with lesional epilepsy also had a higher prevalence of SD. SD was also influenced by the age of epileptic females, the existence of motor weakness, and systolic blood pressure.
11.	Ejigu AK et al. (2019) [21]	The study evaluated SD and related factors in epilepsy patients.	A hospital-based cross-sectional study was conducted among patients with epilepsy. CSFQ-14 was used to assess the SD. 694 respondents participated, with a response rate of 99.14%. Among them, 576 completed the questionnaire out of 363 subjects with global sexual dysfunction. Furthermore, the rate of sexual dysfunction was 55.6% and 67.4% in females and males, respectively.	In people with epilepsy, SD is highly common, and it is more common in men than in women. The most common and least common SD were problems with sexual arousal and pain, respectively.
12.	Lima EM et al. (2021) [22]	Determined the impact of depressive and anxiety symptoms on QOL in epilepsy patients.	The study included 35 patients and 90 healthy controls. QOL was assessed by the ESI and QOLIE-31, and the BDI and the STAI-X were used to assess symptoms of depression and anxiety. Anxiety-trait symptoms are the most critical individual determinant of QOL.	The study concluded that epilepsy patients had greater odds of presenting depressive and anxiety symptoms and worse QOL than healthy controls.
13.	Pinelopi V et al. (2022) [23]	Investigated the effect of the ODTA on the QOL of patients with epilepsy.	15 patients with epilepsy were enrolled in a three-month OTDA program. QOL was measured for patients using the QOLIE-31 mean scores during pre- and post-intervention. Statistically significant improvements were observed in fear of having a seizure (p =.004), overall quality (p =.001), emotional well-being (p =.004), energy fatigue (p =.014), and total QOLIE (p =.001) score after the intervention. The changes in the QOLIE scores were more prominent among female individuals (male vs. female: p =.028 vs. p =.008).	The study concluded that occupational therapy intervention along with ODTA provided statistically significant improvements regarding fear of seizure, overall quality, emotional well-being, energy fatigue, and total QOLIE score after the intervention, and the changes were more prominent among the females.
14.	Sheikhalishahi A et al. (2021) [24]	This study is aimed to investigate the correlation between the sexual function of women with epilepsy and personality factors.	This cross-sectional study was conducted on 100 women with epilepsy to assess SD with women's sexual function index questionnaire and NEO-five-factor of personality inventory. The average score for sexual function among participants was 23.33 ± 2.82. Neuroticism and sexual function showed a significant negative correlation (P = 0.00, r = −0.03), while personality traits such as extraversion (P = 0.00, r = 0.63), agreeableness (P = 0.008, r = 0.26), conscientiousness (P = 0.04, r = 0.20), and openness to new experiences (P = 0.03, r = 0.21) were significantly positively correlated with sexual function in women with epilepsy.	Results indicate that personality traits can affect the sexual health and sexual function of women with epilepsy. In order to prevent SD, it is advised that healthcare professionals evaluate personality traits in women with epilepsy as a predictive factor.
15.	Sureka RK et al. (2021) [25]	The purpose of the study was to evaluate SD in men who were experiencing idiopathic GTCS.	146 male patients with GTCS and a seizure-free period of at least a year were evaluated for SD. The ASEX was used to assess the remaining patients for SD. 66% (n = 66) had a diagnosis of sexual dysfunction. The most prevalent sexual disease identified in 36% of the patients (n = 36) was erectile dysfunction, which was followed by premature ejaculation in 26% of the patients (n = 26) and decreased libido (hypoactive sexual desire) in 4% of the patients (n = 4). A significant correlation was discovered between the duration of epilepsy and the type of treatment (polytherapy versus monotherapy) in individuals with sexual dysfunction (P value < 0.05). Patients receiving polytherapy were more likely to experience erectile dysfunction and hypoactive desire, while those receiving monotherapy were more likely to experience premature ejaculation.	In male epileptics with GTCS, a significant SD was found. A practicing doctor should have a high degree of suspicion while diagnosing and treating SD in them.

Discussion

This review focused on the sexual dysfunction experienced by epileptic patients and noted that these patients frequently experienced dyspareunia, orgasm disorders, and erectile dysfunction. Moreover, decreased sexual desire, early climax/ejaculation, and vaginal dryness ultimately led to a poor quality of life [10]. Sexual behavior is thought to be controlled by the brain. Epilepsy is one of the conditions brought on by brain dysfunction. The clinical characteristic impairment of sexual function varies depending on where the seizures originated. The key contributors to the pathogenesis of sexual dysfunction are abnormalities in sex hormone production. Increasing data suggests that epilepsy may impact either the hypothalamic-pituitary-ovarian or the hypothalamic-pituitary-testicular axis. It is well recognized that bioactive testosterone is essential for both male and female sexual function [10].

Epilepsy patients were found to have significantly lower levels of bioactive testosterone than the healthy controls, which suggests that the epilepsy patients may be hyposexual, which potentially leads to reduced sexual interest and activity. Additionally, sexual dysfunction in male epilepsy patients may be associated with increased follicle-stimulating hormone/sex hormone-binding globulin/prolactin levels as well as decreased GnRH and dehydroepiandrosterone sulfate levels, which can result in hypogonadism. According to a study, sexual dysfunction may also be linked to a decrease in estradiol or dehydroepiandrosterone sulfate in women with epilepsy. It's interesting to note that there appears to be a two-way relationship between seizures and sex hormones, with seizures potentially affecting sex hormone levels and hormones modulating seizures as a result. This suggests a vicious loop between epilepsy and sexual dysfunction [10,26-28].

According to previous research, AED use has been linked to the emergence of sexual hormone abnormalities and sexual dysfunction. On one hand, several AEDs, such as carbamazepine, phenytoin, and phenobarbital, may induce liver enzymes, which directly lead to suppression of gonadal testosterone synthesis and disturbance of other peripheral sex steroid hormones. AEDs may also reduce brain excitability, which may have a detrimental impact on sexuality. Since the natural purpose of AEDs is to control the patients' seizure symptoms, it appears that more sedative AEDs have a greater impact on sexual functioning than those with less sedative effects [10]. Regarding the potential link between AEDs and sexual function, patients and physicians need to consider both seizure status and sexual health when selecting the best AED. According to earlier research, hepatic inducers like phenytoin and carbamazepine incorporate cytochrome p450, modify the levels of sexual hormones, and cause sexual dysfunction. Sexual hormone levels are decreased by phenobarbitone, phenytoin, and carbamazepine, which alter the metabolism of these hormones and increase the hormone-binding globulins. Additionally, it has been demonstrated that phenytoin decreases sperm motility [13,29].

Additionally, epilepsy patients have been linked to symptoms of psychiatric comorbidities such as depression and anxiety. Although the prevalence of anxiety is unknown, that of depression ranges from 11% to 60% [2,30]. According to a study by Souza et al., depression and anxiety are also linked to sexual dysfunction [31]. The search method may have limited the amount of literature included in this study because it only included articles with full-text availability in the English language. As a result, not all of the recent research on sexual dysfunction in epilepsy patients may be represented in the results.

## Conclusions

A high proportion of epilepsy patients are affected by sexual dysfunction. Sexual dysfunction is a common yet underdiagnosed condition in epilepsy patients and has a significant association with other co-morbidities like depression and anxiety, which consequently impact the quality of life in these patients. There is a shortage of studies examining the association of sexual dysfunction with depression, anxiety, and newer non-enzyme-inducing drugs. The high proportion of cases found to have sexual dysfunction in epilepsy patients warrants an urgent need for physicians to be aware of this aspect of epilepsy. Epilepsy patients often do not self-report this problem due to the stigma attached to it. Hence, physicians and neurologists must keep a high index of suspicion for this problem. Furthermore, screening and monitoring for sexual dysfunction should be added to the usual epilepsy work-up.
